# Supplementary material for: Awareness and Perceptions among Members of a Japanese Cancer Patient Advocacy Group Concerning the Financial Relationships between the Pharmaceutical Industry and Physicians
Source: Int J Environ Res Public Health. 2022 Mar 15;19(6):3478. doi: 10.3390/ijerph19063478 (PMC8952770; doi:10.3390/ijerph19063478)
Supplement: Supplementary file 1 [file ijerph-19-03478-s001.zip › ijerph-1562140-supplementary.pdf]

Supporting information:

Supplementary Material S1 (Supplementary Methods)

Variables

Sociodemographic factors. Sociodemographic factors included age, sex, highest educational attainment (*less than high school graduate; high school graduate; associate degree or diploma; or bachelor's degree or more*), employment status (*full time job; self-employed; part-time job; unemployed; or other*), and annual income (*<2 million JPY (<18,349 USD); 2-4 million JPY (18,349-36,697 USD); 4-6 million JPY (36,697-55,046 USD); 6-8 million JPY (55,046-73,394 USD); 8-10 million JPY (73,394-91,743 USD); or >10 million JPY (>91,743 USD*).

Primary diagnosis. We asked about the participant's primary disease and categorized it into seven types: *non-cancer, prostate cancer, lung cancer, breast cancer, colorectal cancer, gastric cancer, and other kinds of cancer*.

Clinical factors relating to cancer (only for cancer patients). For cancer patients, we collected data on clinical characteristics including disease stage (*Stage I, Stage II, Stage III, Stage IV, or unclear*), year of diagnosis (*2018, 2017, 2016, 2015, or before 2015*), type of hospital in which participant received treatment (*Cancer hospital, national university hospital, private university hospital, national municipal hospital, private municipal hospital, or other hospital*), the treatment participants had been receiving (*no active treatment, anticancer drug, molecularly targeted drug, immune checkpoint inhibitor, hormonal therapy, radiation therapy, or other therapy*), treatments participants had ever received (*no active treatment, anticancer drug, molecularly targeted drug, immune checkpoint inhibitor, hormonal therapy, radiation therapy, surgery, or other therapy*), and disease recurrence (*yes, no, not clear, or other*).

Awareness of physician-Pharma interactions. To assess awareness of physician-Pharma interactions, we evaluated awareness of 11 types of interactions, including pamphlets and leaflets, stationeries, textbooks and articles, free drug samples, free meals travel and accommodations, lecture fees, disclosure program of the payment from Pharma to physicians, conduct of research for Pharma, outsourcing expenses, and possession of stocks. Consequently, we used the following question, *"Do you know that some physicians would receive pamphlets and leaflets concerning products manufactured by pharmaceutical companies from their sales representatives?"* on a three-point Likert scale (*Yes, No, or Not sure*). Other items are

listed in Supplementary Material S2.

Influence on trust in physicians. We assessed the impact on trust in physicians over 11 types of interactions including, pamphlets and leaflets, stationeries, textbooks and articles, free drug samples, free meals, travel and accommodations, lecture fees, conduct of research for Pharma, outsourcing expenses, possession of stocks, and insider trading. Consequently, we used the following question, *"How would your trust in your physician be influenced if your physician received pamphlet or leaflet with information about the products from a pharmaceutical sales representative?"* on a five-point Likert scale (*Increase of trust in physicians, Slight increase of trust in physicians, Neither increase nor decrease of trust in physicians, Slight decrease of trust in physicians, or Decrease of trust in physicians*). Other items are listed in Supplementary Material 3.

Perceptions on physician-Pharma interactions. We evaluated participants' perception of physician-Pharma interactions by assessing whether to agree with the following statements. 1) *"Payments from pharmaceutical companies would influence physician prescription pattern."* 2) *"Payments from pharmaceutical companies would increase unnecessary prescriptions and healthcare costs."* 3) *"A receipt of payments from pharmaceutical companies would be acceptable as long as its amount is small."* 4) *"Physician's receipts of gifts, meals and honoraria from pharmaceutical companies are unethical."* 5) *"Physician's receipts of gifts, meals, and honoraria from pharmaceutical companies would undermine the trust in physicians."* 6) *"More rigorous internal regulation of gifts, meals, and honoraria from pharmaceutical companies to physicians is needed among pharmaceutical companies."* 7) *"More rigorous internal regulation of gifts, meals, and honoraria from pharmaceutical companies to physicians is needed among physicians."* 8) *"More rigorous legal regulation of gifts, meals, and honoraria from pharmaceutical companies to physicians is needed."* On a five-point Likert scale (*Agree, Slightly agree, Neither agree nor disagree, Slightly disagree, or Disagree*). Further, for participants who agree or slightly agree with the third statement, we additionally asked the closet amount of money which they think is acceptable on a four-point scale (*1,000 JPY (9.2 USD) or below, 1,000-3,000 JPY (9.2-27.5 USD), 3,000-10,000 JPY (27.5-91.7 USD), or other*). For participants who agreed or slightly agreed with either of sixth, seventh or eighth statement, we additionally asked the monetary value (*100 thousand JPY (917 USD) or below, 100 thousand-1 million JPY (917-9174 USD), 1-5 million JPY (9,174-45,872 USD), 5-10 million JPY (45,872-91,743 USD, or 10-30 million JPY (91,743-275,229 USD)*) and frequency (*twice a week, once a week, once every two weeks, once a month, or once every few months*) of the financial

interaction with the Pharma. (Supplementary Material S4)

Attitude towards various professional FCOI. Attitude towards various professional FCOI was assessed by using the following five statements. 1) *"It is problematic for judges to receive gifts, meals, and other entertainment from the trial attorney in charge."* 2) *"It is problematic for sports referees to receive gifts, meals, and other entertainment from the players of the games they are assigned to."* 3) *"It is problematic for politicians to receive gifts, meals, and other entertainment from lobbyists."* 4) *"It is problematic for physicians to receive gifts, meals, and other entertainment from pharmaceutical sales representatives."* 5) *"It is problematic for business people to receive gifts, meals, and other entertainment from their customers."* On a five-point Likert scale (*Agree, Slightly agree, Neither agree nor disagree, Slightly disagree, or Disagree*).

An open-ended question about participants' perception of FCOI between Pharma and physicians. To dig deeper into participants' perceptions on FCOI between Pharma and physicians, we investigated responses to an open-ended question: "*Please freely describe your thoughts about non-research-related offerings from Pharma to a physician (e.g., gifts, free meals, monetary incentives for a lecture).*" The responses to this question were analyzed separately through qualitative analysis.

Supplementary Material S2. List of questions regarding awareness of physician-Pharma interactions.

	Question	Response options
**1**	Do you know that some physicians would receive pamphlets and leaflets concerning products manufactured by pharmaceutical companies from their sales representatives?	Yes<br>No<br>Not sure
**2**	Do you know that some physicians would receive stationeries embedded with pharmaceutical company names from pharmaceutical companies?	Yes<br>No<br>Not sure
**3**	Do you know that some physicians would receive textbooks and academic articles for daily practice from pharmaceutical companies?	Yes<br>No<br>Not sure
**4**	Do you know that some physicians would receive free drug samples from pharmaceutical companies?	Yes<br>No<br>Not sure
**5**	Do you know that some physicians would receive free meals from pharmaceutical companies at study meetings and conferences?	Yes<br>No<br>Not sure
**6**	Do you know that some physicians would receive travel and accommodation reimbursements from pharmaceutical companies to attend conferences?	Yes<br>No<br>Not sure
**7**	Do you know that some physicians would receive 10-100 thousand JPY (about 100-1000 USD) as honoraria from pharmaceutical companies when they serve as lecturers at conferences held by pharmaceutical companies?	Yes<br>No<br>Not sure
**8**	Do you know that pharmaceutical companies disclose payments made to healthcare professionals and healthcare organizations for promotional and other purposes?	Yes<br>No<br>Not sure
**9**	Do you know that some physicians would participate in researches sponsored by pharmaceutical companies?	Yes<br>No<br>Not sure

**10**	Do you know that some healthcare organizations receive several hundred thousand JPY of honoraria from pharmaceutical companies in exchange for enrolment of their patients for treatment in clinical trials sponsored by pharmaceutical companies?	Yes<br>No<br>Not sure
**11**	Do you know some physicians would have stock in pharmaceutical companies?	Yes<br>No<br>Not sure

Supplementary Material S3. List of questions regarding influence on trust in physicians

	Question	Response options
**1**	How would your trust in your physician be influenced if your physician received pamphlets or leaflets with information about the products from a pharmaceutical sales representative?	Increase in trust of physicians<br>Slight increase in trust of physicians<br>Neither increase nor decrease in trust of physicians<br>Slight decrease in trust of physicians<br>Decrease in trust of physicians
**2**	How would your trust in your physician be influenced if your physician received stationeries with a pharmaceutical company name from pharmaceutical companies?	Increase in trust of physicians<br>Slight increase in trust of physicians<br>Neither increase nor decrease in trust of physicians<br>Slight decrease in trust of physicians<br>Decrease in trust of physicians
**3**	How would your trust in your physician be influenced if your physician received textbooks or articles concerning daily clinical practice from pharmaceutical companies?	Increase in trust of physicians<br>Slight increase in trust of physicians<br>Neither increase nor decrease in trust of physicians<br>Slight decrease in trust of physicians<br>Decrease in trust of physicians
**4**	How would your trust in your physician be influenced if your physician received free drug samples from pharmaceutical companies?	Increase in trust of physicians<br>Slight increase in trust of physicians

		Neither increase nor decrease in trust of physicians<br>Slight decrease in trust of physicians<br>Decrease in trust of physicians
**5**	How would your trust in your physician be influenced if your physician received free meals from pharmaceutical companies at training sessions and lecture meetings?	Increase in trust of physicians<br>Slight increase in trust of physicians<br>Neither increase nor decrease in trust of physicians<br>Slight decrease in trust of physicians<br>Decrease in trust of physicians
**6**	How would your trust in your physician be influenced if your physician received travel and accommodation reimbursement from pharmaceutical companies to attend lecture meetings?	Increase in trust of physicians<br>Slight increase in trust of physicians<br>Neither increase nor decrease in trust of physicians<br>Slight decrease in trust of physicians<br>Decrease in trust of physicians
**7**	How would your trust in your physician be influenced if your physician received 10-100 thousand JPY (about 100-1000 USD) as lecture fees from pharmaceutical companies for lecturing about its drugs on behalf of pharmaceutical companies at meetings?	Increase in trust of physicians<br>Slight increase in trust of physicians<br>Neither increase nor decrease in trust of physicians<br>Slight decrease in trust of physicians<br>Decrease in trust of physicians
**8**	How would your trust in your physician be influenced if your physician conducted research sponsored by pharmaceutical companies?	Increase in trust of physicians<br>Slight increase in trust of physicians<br>Neither increase nor decrease in trust of physicians

		Slight decrease in trust of physicians<br>Decrease in trust of physicians
**9**	How would your trust in your physician be influenced if your healthcare organization received several hundred thousand JPY honoraria from pharmaceutical companies to enroll their patients for treatment in a clinical trial sponsored by a pharmaceutical company?	Increase in trust of physicians<br>Slight increase in trust of physicians<br>Neither increase nor decrease in trust of physicians<br>Slight decrease in trust of physicians<br>Decrease in trust of physicians
**10**	How would your trust in your physician be influenced if your physician had stock in pharmaceutical companies?	Increase in trust of physicians<br>Slight increase in trust of physicians<br>Neither increase nor decrease in trust of physicians<br>Slight decrease in trust of physicians<br>Decrease in trust of physicians
**11**	How would your trust in your physician be influenced if your physician knew the promising new drug before the information became public and purchased the stock in the pharmaceutical company?	Increase in trust of physicians<br>Slight increase in trust of physicians<br>Neither increase nor decrease in trust of physicians<br>Slight decrease in trust of physicians<br>Decrease in trust of physicians

Supplementary Material S4. List of questions regarding perception on physician-Pharma interactions.

	Question	Response options
**1**	Payments from pharmaceutical companies would influence physician prescription patterns.	Agree<br>Slightly agree<br>Neither agree nor disagree<br>Slightly disagree<br>Disagree
**2**	Payments from pharmaceutical companies would increase unnecessary prescriptions and healthcare costs.	Agree<br>Slightly agree<br>Neither agree nor disagree<br>Slightly disagree<br>Disagree
**3**	Receipt of payments from pharmaceutical companies would be acceptable if the amount is small.	Agree<br>Slightly agree<br>Neither agree nor disagree<br>Slightly disagree<br>Disagree
**4**	Physicians' receipt of gifts, meals, and honoraria from pharmaceutical companies is unethical.	Agree<br>Slightly agree<br>Neither agree nor disagree<br>Slightly disagree<br>Disagree
**5**	Physicians' receipt of gifts, meals, and honoraria from pharmaceutical companies would undermine the trust in physicians.	Agree<br>Slightly agree<br>Neither agree nor disagree<br>Slightly disagree<br>Disagree
**6**	More rigorous internal regulation of gifts, meals, and honoraria from pharmaceutical companies to physicians is needed among pharmaceutical companies.	Agree<br>Slightly agree<br>Neither agree nor disagree

		Slightly disagree<br>Disagree
**7**	More rigorous internal regulation of gifts, meals, and honoraria from pharmaceutical companies to physicians is needed among physicians.	Agree<br>Slightly agree<br>Neither agree nor disagree<br>Slightly disagree<br>Disagree
**8**	More rigorous legal regulation of gifts, meals, and honoraria from pharmaceutical companies to physicians is needed.	Agree<br>Slightly agree<br>Neither agree nor disagree<br>Slightly disagree<br>Disagree
**9**	For participants who agreed or slightly agreed with the third statement, what is the closest amount of money that you consider as a small amount?	1,000 JPY or below<br>1,000-3,000 JPY<br>3,000-10,000 JPY<br>Other
**10**	For participants who agreed or slightly agreed with either the sixth, seventh or eighth statements, what is the closest thing to the frequency of non-research benefits from pharmaceutical companies to physicians that you consider acceptable?	100 thousand JPY or below<br>100 thousand-1 million JPY<br>1-5 million JPY<br>5-10 million JPY<br>10-30 million JPY
**11**	For participants who agreed or slightly agreed with either the sixth, seventh or eighth statements, what is the closest approximation to the annual total amount of non-research benefits from pharmaceutical companies to physicians that you consider to be acceptable?	Twice a week<br>Once a week<br>Once every two weeks<br>Once a month<br>Once every few months

Supplementary Material S5. Demographic breakdown of participants aware of at least one physician-Pharma interaction.

Variables	Number (%)		*P* value
	Aware	Unaware or not sure	
**Gender**			
**Male**	49 (77.8)	14 (22.2)	0.053
**Female**	28 (93.3)	2 (6.7)	
**Age category**			
**≦60**	13 (86.7)	2 (13.3)	0.879
**61-70**	24 (80.0)	6 (20.0)	
**≥71**	39 (79.6)	10 (20.4)	
**Income**			
**Lower income (<4 million JPY (<36,697 USD))**	39 (84.8)	7 (15.2)	0.129
**Higher income (≥4 million JPY (≥36,697 USD))**	29 (72.5)	11 (27.5)	
**Job**			
**Employed**	30 (81.1)	7 (18.9)	0.576
**Unemployed**	45 (80.4)	11 (19.6)	
**Education**			
**High school graduate or less**	21 (75.0)	7 (25.0)	0.264
**Associate degree or more**	54 (83.1)	11 (16.9)	
**Cancer**			
**Non-cancer participants**	17 (85.0)	3 (15.0)	0.433
**Cancer patients**	55 (79.7)	14 (20.3)	
**Cancer stage[1]**			
**1**	17 (73.9)	6 (26.1)	0.287
**2-4**	26 (83.9)	5 (16.1)	
**Year[1]**			
**2015-2018**	15 (88.2)	2 (11.8)	0.320
**Before 2015**	41 (78.9)	11 (21.1)	
**Hospital[1]**			
**Other hospitals**	24 (80.0)	6 (20.0)	0.555
**Cancer special hospitals**	31 (81.6)	7 (18.4)	
**Previous cancer recurrence[1]**			
**No or other**	41 (78.9)	11 (21.1)	0.246
**Yes**	12 (92.3)	1 (7.7)	
**Experience with pharmacotherapy[1]**			
**No**	19 (73.1)	7 (26.9)	0.145
**Yes**	33 (86.8)	5 (13.2)	
**Experience with radiotherapy[1]**			
**No**	23 (85.2)	4 (14.8)	0.447
**Yes**	29 (80.6)	7 (19.4)	
**Previous surgical treatment[1]**			
**No**	22 (75.9)	7 (25.1)	0.292
**Yes**	32 (84.2)	6 (15.8)	

[1]The analysis included only participants with cancer. Abbreviations: JPY; Japanese yen, USD; US dollar, Pharma; Pharmaceutical Company.

Supplementary Material S6. Logistic regression analysis of participants' awareness of physician-Pharma interactions.

Variables	Odds ratio (95% confidence interval)	*P* value
**Gender**		
**Male**	Ref.	
**Female**	4.00 (0.85–18.90)	0.080
**Age category**		
**≦60**	Ref.	
**61-70**	0.62 (0.11–3.49)	0.584
**≥71**	0.60 (0.12–3.10)	0.542
**Income**		
**Lower income (<4 million JPY)**	Ref.	
**Higher income (≥4 million JPY)**	0.47 (0.16–1.37)	0.168
**Job**		
**Employed**	Ref.	
**Unemployed**	0.95 (0.33–2.74)	0.931
**Education**		
**High school graduate or less**	Ref.	
**Associate degree or more**	1.64 (0.56–4.79)	0.369
**Cancer**		
**Non-cancer participants**	Ref.	
**Cancer patients**	0.69 (0.18–2.70)	0.598
**Cancer stage**[1]		
**1**	Ref.	
**2-4**	1.84 (0.48–6.97)	0.373
**Year**[1]		
**2015-2018**	Ref.	
**Before 2015**	0.50 (0.10–2.51)	0.397
**Hospital**[1]		
**Other hospitals**	Ref.	
**Cancer special hospitals**	1.11 (0.33–3.73)	0.869
**Previous cancer recurrence**[1]		
**No or other**	Ref.	
**Yes**	3.22 (0.38–27.52)	0.286
**Experience with pharmacotherapy**[1]		
**No**	Ref.	
**Yes**	2.43 (0.68–8.74)	0.173
**Experience with radiotherapy**[1]		
**No**	Ref.	
**Yes**	0.72 (0.19–2.76)	0.633
**Previous surgical treatment**[1]		
**No**	Ref.	
**Yes**	1.70 (0.50–5.74)	0.395

[1]The analysis included only participants with cancer. Abbreviations: JPY; Japanese yen, Pharma; Pharmaceutical Company, Ref; reference value.

Supplementary Material S7. Number and percent of participants reporting decreased trust in at least one physician-Pharma interaction by participant demographics.

Variables	Number (%)		*P* value
	Decrease trust	Other	
**Gender**			
**Male**	55 (87.3)	8 (12.7)	0.157
**Female**	28 (96.6)	1 (3.4)	
**Age category**			
**≦60**	14 (93.3)	1 (6.7)	0.550
**61-70**	26 (89.7)	3 (10.3)	
**≥71**	44 (89.8)	5 (10.2)	
**Income**			
**Lower income (<4 million JPY)**	41 (89.1)	5 (10.9)	0.590
**Higher income (≥4 million JPY)**	36 (90.0)	4 (10.0)	
**Job**			
**Employed**	33 (98.2)	4 (10.8)	0.515
**Unemployed**	51 (91.1)	5 (8.9)	
**Education**			
**High school graduate or less**	23 (82.1)	5 (17.7)	0.089
**Associate degree or more**	61 (93.9)	4 (6.1)	
**Cancer**			
**Non-cancer participants**	19 (95.0)	1 (5.0)	0.424
**Cancer patients**	62 (89.9)	7 (10.1)	
**Cancer stage**[1]			
**1**	22 (95.7)	1 (4.3)	0.283
**2-4**	27 (87.1)	4 (12.9)	
**Year**[1]			
**2015-2018**	14 (82.4)	3 (17.6)	0.316
**Before 2015**	46 (90.2)	5 (9.8)	
**Hospital**[1]			
**Other hospitals**	26 (89.7)	3 (10.3)	0.517
**Cancer special hospitals**	33 (86.8)	5 (13.2)	
**Previous cancer recurrence**[1]			
**No or other**	47 (90.4)	5 (9.6)	0.428
**Yes**	11 (84.6)	2 (15.4)	
**Experience with pharmacotherapy**[1]			
**No**	24 (92.3)	2 (7.7)	0.398
**Yes**	33 (86.8)	5 (13.2)	
**Experience with radiotherapy**[1]			
**No**	25 (92.6)	2 (7.4)	0.349
**Yes**	31 (86.1)	5 (13.9)	
**Previous surgical treatment**[1]			
**No**	24 (82.8)	5 (17.2)	0.119
**Yes**	36 (94.7)	2 (5.3)	

[1]The analysis included only participants with cancer. Abbreviations: JPY; Japanese yen, Pharma; Pharmaceutical Company.

Supplementary Material S8. Logistic regression analysis of the influence of physician-Pharma relationships on participants' trust.

Variables	Odds ratio (95% confidence interval)	*P* value
**Gender**		
**Male**	Ref.	
**Female**	4.07 (0.48–34.21)	0.185
**Age category**		
**≦60**	Ref.	
**61-70**	0.62 (0.059–6.52)	0.713
**≥71**	0.63 (0.068–5.84)	0.683
**Income**		
**Lower income (<4 million JPY)**	Ref.	
**Higher income (≥4 million JPY)**	1.10 (0.27–4.40)	0.895
**Job**		
**Employed**	Ref.	
**Unemployed**	1.24 (0.31–4.94)	0.764
**Education**		
**High school graduate or less**	Ref.	
**Associate degree or more**	3.32 (0.82–13.44)	0.093
**Cancer**		
**Non-cancer participants**	Ref.	
**Cancer patients**	0.47 (0.054–4.03)	0.488
**Cancer stage[1]**		
**1**	Ref.	
**2-4**	0.31 (0.032–2.95)	0.306
**Year[1]**		
**2015-2018**	Ref.	
**Before 2015**	1.97 (0.42–9.30)	0.376
**Hospital[1]**		
**Other hospitals**	Ref.	
**Cancer special hospitals**	0.76 (0.17–3.48)	0.689
**Previous cancer recurrence[1]**		
**No or other**	Ref.	
**Yes**	0.59 (0.10-3.42)	0.552
**Experience with pharmacotherapy[1]**		
**No**	Ref.	
**Yes**	0.55 (0.098–3.08)	0.496
**Experience with radiotherapy[1]**		
**No**	Ref.	
**Yes**	0.50 (0.089–2.78)	0.425
**Previous surgical treatment[1]**		
**No**	Ref.	
**Yes**	3.75 (0.67–20.93)	0.132

[1] The analysis included only participants with cancer. There were no statistically significant differences between the influence of physician-pharmaceutical company relationships on participants' trust and each variable. Abbreviations: JPY; Japanese yen, Pharma; Pharmaceutical Company.
